# Safety and effectiveness of eculizumab for pediatric patients with atypical hemolytic–uremic syndrome in Japan: interim analysis of post-marketing surveillance

**DOI:** 10.1007/s10157-018-1610-2

**Published:** 2018-07-23

**Authors:** Shuichi Ito, Yoshihiko Hidaka, Norimitsu Inoue, Shinya Kaname, Hideki Kato, Masanori Matsumoto, Yoshitaka Miyakawa, Masashi Mizuno, Hirokazu Okada, Akihiko Shimono, Takahisa Matsuda, Shoichi Maruyama, Yoshihiro Fujimura, Masaomi Nangaku, Shoji Kagami

**Affiliations:** 1 Department of Pediatrics, Graduate School of Medicine, Yokohama City University, 3-9 Fukuura, Kanazawa-ku, Yokohama, Kanagawa 236-0004 Japan; 2 Clinical Division of Pediatrics, Shinshu University Hospital, 3-1-1 Asahi, Matsumoto, Nagano 390-8621 Japan; 3 Department of Tumor Immunology, Osaka International Cancer Institute, 3-1-69 Otemae, Chuo-ku, Osaka, 541-8567 Japan; 4 Department of Nephrology and Rheumatology, Kyorin University School of Medicine, 6-20-2 Shinkawa, Mitaka, Tokyo 181-8611 Japan; 5 Division of Nephrology and Endocrinology, The University of Tokyo, 7-3-1, Hongo, Bunkyo-ku, Tokyo, 113-8655 Japan; 6 Department of Blood Transfusion Medicine, Nara Medical University, 840 Shijyo-cho, Kashihara, Nara 634-8522 Japan; 7 Department of General Internal Medicine, Thrombosis and Hemostasis Center, Saitama Medical University Hospital, 38 Moroyama, Iruma-gun, Saitama 350-0495 Japan; 8 Department of Nephrology, Nagoya University Graduate School of Medicine, 65 Tsurumai-cho, Showa-ku, Nagoya, Aichi 466-8550 Japan; 9 Saitama Medical University, 38 Moroyama, Iruma-gun, Saitama 350-0495 Japan; 10 Alexion Pharma GK, 1-18-14 Ebisu, Shibuya-ku, Tokyo, 150-0013 Japan; 11 Japanese Red Cross Kinki Block Blood Center, 7-5-17, Saitoasagi, Ibaraki, Osaka 567-0085 Japan; 12 Department of Pediatrics, Institute of Health Biosciences, The University of Tokushima Graduate School, Kuramoto cho 3-chome, Tokushima, 770-8503 Japan

**Keywords:** Atypical hemolytic–uremic syndrome, Post-marketing surveillance, Complement protein C5 inhibitor, Eculizumab

## Abstract

**Background:**

In 2013, eculizumab was approved for treatment of the atypical hemolytic–uremic syndrome (aHUS) in Japan, which was defined as a thrombotic microangiopathy (TMA) excluding Shiga toxin-producing *Escherichia coli-*HUS and thrombotic thrombocytopenic purpura. Simultaneously, post-marketing surveillance was started to assess its safety and effectiveness. In 2016, Japanese clinical guide redefined terms to limit the use of “aHUS” to complement-mediated HUS only. Accordingly, TMA with other causes was defined as secondary TMA. Here we report the interim analysis of post-marketing surveillance of pediatric patients with aHUS and secondary TMA.

**Methods:**

Pediatric patients treated with eculizumab from approval to 15 March 2017 were included in this observational real-world study. Clinical endpoints of effectiveness were TMA event–free status, complete TMA response, platelet count normalization, and improvement of estimated glomerular filtration rate (eGFR). Adverse reactions to eculizumab were also analyzed.

**Results:**

In 27 pediatric patients with aHUS, median age at diagnosis was 4 years. Complement genes’ variants were detected in 14 of 21 patients (66.7%). Median time from diagnosis to eculizumab initiation was 2.0 days. TMA event–free status, complete TMA response, platelet normalization, and improvement in eGFR were achieved in 85.2, 36.4, 78.3, and 75.0% of patients, respectively. Three patients with aHUS died. Twenty-four and 10 adverse reactions were reported in 31 aHUS patients and 17 secondary TMA patients, respectively; however, no eculizumab-related death or meningococcal infection was reported.

**Conclusions:**

This interim analysis confirmed that eculizumab is well-tolerated and effective for Japanese pediatric patients with aHUS in a real-world setting.

**Electronic supplementary material:**

The online version of this article (10.1007/s10157-018-1610-2) contains supplementary material, which is available to authorized users.

## Introduction

Atypical hemolytic–uremic syndrome (aHUS), a thrombotic microangiopathy (TMA), is a rare disease characterized by the triad of microangiopathic hemolytic anemia (MAHA), thrombocytopenia, and acute kidney injury (AKI) [1, 2]. The prognosis of aHUS was very poor: 29–48% of children with aHUS progressed to end-stage renal disease or death within 1 year when the mainstay of treatment was plasma therapy [3, 4].

Eculizumab (Soliris®, Alexion Pharmaceuticals) is a recombinant humanized monoclonal antibody against complement protein 5 (C5). Based on results from clinical studies [5, 6], it was approved for treatment of aHUS in Japan in September 2013 [7]. Eculizumab binds C5 and blocks cleavage of C5 to C5a and C5b, thus inhibiting formation of the terminal complement complex. Pivotal clinical studies showed that eculizumab significantly inhibited complement-mediated TMA and improved renal function in a time-dependent manner [6]. Since aHUS is rare, and data on its safety and effectiveness are limited, especially in Japanese, the Ministry of Health, Labour and Welfare of Japan requested Alexion Pharma GK to monitor all aHUS patients treated with eculizumab, as a condition for approval. In September 2013, regulatory mandated post-marketing surveillance (PMS) was started to assess the long-term safety and effectiveness of eculizumab for all patients treated in Japan and will be completed by July 2018.

The diagnostic criteria for aHUS were defined in a Japanese clinical guide by the Japan Pediatric Society and Japanese Society of Nephrology, published in 2013 [8, 9]. In these guides, aHUS was broadly defined as any TMA excluding Shiga toxin–producing *Escherichia coli* (STEC)-HUS and thrombotic thrombocytopenic purpura (TTP) [8, 9]. The definition of aHUS has changed over time in Japan; therefore, the revised guide 2015 (published in 2016) limits the use of the term “aHUS” to complement-mediated HUS only, thereby excluding any TMA associated with transplantation, infection, drugs, autoimmune diseases, malignancies, or metabolic disorders (which is defined as “secondary TMA”) [2]. Nucleotide sequencing and analysis of complement genes were recommended; however, the identification of a pathogenic mutation is not always required for a diagnosis of aHUS [2]. Throughout this report, we use “aHUS” to mean complement-mediated HUS, according to the definition in the 2015 Japanese clinical guide. However, both aHUS and secondary TMA are included in the present analysis, because the broader (i.e., 2013) definition of aHUS was used for diagnosis in the patient population included.

The primary objective of this PMS is to collect data on the safety and effectiveness of long-term eculizumab treatment for all patients diagnosed with aHUS or secondary TMA who received at least one dose of eculizumab in Japan. Here, we report the real-world data in an interim analysis.

## Methods

### Study design and patients

The requirements of ethical approval by an institutional review board and informed consent from individual patients were waived for this regulatory mandated observational study. Patients younger than 18 years were enrolled if they had received an aHUS diagnosis from patient’s physician and had used eculizumab according to Japanese clinical guides [2, 8, 9] during the period from September 2013 to March 2017. The inclusion criteria were presence of MAHA, thrombocytopenia, and AKI; patients with STEC-HUS or TTP were excluded [8, 9]. MAHA was defined as a hemoglobin level of < 10 g/dL and thrombocytopenia as a platelet count (PLT) of < 15 × 10^4^/µL. AKI in children was defined as a serum creatinine level at least 1.5 times the upper limit of the age- and sex-specific pediatric reference range [10]. The approved eculizumab dosing is based on the patient’s weight (Supplementary Table 1) [7].

### Assessments of effectiveness and safety

The clinical endpoints of effectiveness, TMA event–free status, complete TMA response, hematologic outcomes, and renal outcomes, and the definitions of adverse events (AEs) and adverse reactions (ARs) of eculizumab were described in Supplementary Table 2.

### Statistical analysis

Descriptive analysis of patient characteristics at the start of eculizumab treatment (baseline) was performed using median, mean, standard deviation (SD), and range (for continuous variables) and frequency and proportions (for categorical variables). Safety was summarized as the numbers of patients and incidence rates (in person-years) for each event in the safety analysis sets for aHUS and secondary TMA, respectively.

In the effectiveness analysis, clinical endpoints of effectiveness during treatment were indicated by the numbers and proportions of patients with aHUS who achieved the endpoint of interest. Absolute values and changes from baseline in PLT, lactate dehydrogenase (LDH), and eGFR were summarized using descriptive statics. Missing data were not imputed, except for body weight at the time of eculizumab administration, which was imputed using the most recent data before administration. Statistical analyses were performed with SAS version 9.1.3 (SAS Institute, Cary, NC). Two-sided *P* values (significance level 0.05) were used in all analyses.

## Results

### Pediatric patients enrolled in PMS

Forty-eight pediatric patients (31 with aHUS and 17 with secondary TMA) were enrolled. The meningococcal vaccine was administered to 54.2% of patients (26/48), 16 (61.5%, 16/26) received the vaccine after initiation of eculizumab treatment. Four patients (15.4%, 4/26) received prophylactic antibiotic therapy. Four patients were excluded from the effectiveness analysis, because they started eculizumab before drug approval and some data were thus not collected at baseline. Therefore, 44 pediatric patients—27 with aHUS and 17 with secondary TMA—were included in the effectiveness analysis.

### Characteristics of patients with aHUS (complement-mediated HUS)

The characteristics of the 27 aHUS patients are summarized in Table 1. Median age (range) at the start of eculizumab treatment was 4 (0–16) years, and 11 (40.7%) patients were younger than 1 year. Median weight (range) was 14.9 (4.3–52.0) kg. DNA sequences of complement genes were examined in 21 patients; of these patients, genetic variants or autoantibodies were identified in 14 patients (66.7%), and 2 or more mutations/polymorphisms were found in 6 patients. Identified variants with an allele frequency of < 0.005 in either ExAC or HGVD databases are summarized in Supplementary Table 3. Anti-CFH antibodies were detected in 3 patients, all of whom had *CFHR1*/*3* deletions.

**Table 1:** Baseline demographics and disease characteristics of patients with aHUS (*n* = 27)

Valuable	
Median age at 1st eculizumab administration, years (range), *n* = 27	4.0 (0–16)
1 month– < 23 months, *n* (%)	11 (40.7)
≥ 23 months– < 5 years, *n* (%)	4 (14.8)
≥ 5–< 12 years, *n* (%)	9 (33.3)
≥ 12–< 18 years, *n* (%)	3 (11.1)
Median weight, kg (range), *n* = 27	14.90 (4.3–52.0)
Female sex, *n* (%)	10 (37.0)/27
Patient reported family history of aHUS, *n* (%)	3 (11)/27
Identified complement gene mutation, autoantibody or polymorphism, *n* (%)/examined	14 (66.7)/21
One mutation/polymorphism	8 (57.1)
Two or more mutations/polymorphisms^a^	6 (42.9)
*C3*, *n* (%)	3 (21.4)
*CFB*, *n* (%)	2 (14.3)
*CFH*, *n* (%)	4 (28.6)
*CFHR1/3* deletion, *n* (%)	4 (28.6)
anti-CFH antibody, *n* (%)	3 (21.4)
*CFI*, *n* (%)	0 (0.0)
*MCP*, *n* (%)	5 (35.7)
*DGKE*, *n* (%)	1 (7.1)
Other genes^b^	1 (7.1)
Median period from 1st TMA symptom to the first administration of eculizumab, (days) median (range), *n* = 27	14.0 (2–1692)
Median period from the day of diagnosis to the first administration of eculizumab (days), median (range), *n* = 27	2.0 (1–1316)
Plasma therapy (past 1 year), *n* (%)	19 (70.4)/27
Median days of plasma therapy from the closest TMA to diagnosis to the first administration of eculizumab (days), median (range), *n* = 27	3.0 (0–53)
Dialysis at diagnosis (past 1 year), *n* (%)	13 (48.1)/27
Previous renal transplant, *n* (%)	0 (0.0)/27
Mean platelet count, × 10^4^/µL, (SD), *n* = 27	8.89 (9.24)
Platelet count < 15 × 10^4^/µL, *n* (%)	23 (85.2)/27
Mean LDH level, U/l (SD), *n* = 27	1315.4 (1045.5)
LDH greater than ULN, *n* (%)	25 (92.6)/27
Mean hemoglobin concentration, g/dl (SD), *n* = 27	8.63 (2.62)
Hemoglobin concentration < 10 g/dl, *n* = 27	24 (88.9)
Schistocytes positive, *n* (%)/examined	6 (100)/6
Mean serum creatinine level, mg/dl (SD), *n* = 27	1.73 (1.63)
Mean eGFR, ml/min/1.73 m^2^ (SD), *n* = 15	40.44 (40.32)
eGFR (ml/min/1.73 m^2^), *n*	15
< 15, *n* (%)	4 (26.7)
15–29, *n* (%)	6 (40.0)
30–44, *n* (%)	1 (6.7)
45–59, *n* (%)	1 (6.7)
60–89, *n* (%)	0 (0.0)
≥ 90, *n* (%)	3 (20.0)
Median duration of eculizumab treatment, weeks (range), *n* = 27	51.0 (0–125)
< 1 week, *n* (%)	2 (7.4)
≥ 1 week, < 4 weeks, *n* (%)	2 (7.4)
≥ 4 weeks, < 26 weeks, *n* (%)	3 (11.1)
≥ 26 weeks, *n* (%)	20 (74.1)

*C3* Complement component 3, *CFB* complement factor B, *CFH* complement factor H, *CFHR* CFH-related protein, *CFI* complement factor I, *MCP* membrane cofactor protein, *DGKE* diacylglycerol kinase ε, *CFHR1/3* denotes the locus from the CFHR3 to the CFHR1 genes, *TMA* thrombotic microangiopathy, *LDH* lactate dehydrogenase, *ULN* upper limit of normal, *eGFR* estimated glomerular filtration rate

^a^The combinations of identified genetic variants were [C3- p.Ile1157Thr and MCP- p.Pro195Ser], [CFB- p.Leu9His and MCP- p.Ala311Val], [CFH- p.Phe176Leu- p.Arg1215Gln and CFHR5- p.Pro453Ser], [C3- p.Ser179Pro and CFH- p.His402Tyr- p.Glu936Asp], [CFH- p.His402Tyr- p.Glu936Asp and MCP- p.Thr163Ile], and [DGKE-c.71delT and -c.1213-2A > G]. CFH- p.His402Tyr and p.Glu936Asp are common variants in Japanese

^b^CFHR5 variant p.Pro453Ser

The median interval (range) from first TMA manifestation and aHUS diagnosis to the first dose of eculizumab was 14.0 (2–1692) days and 2.0 (1–1316) days, respectively (Table 1). Twenty-five of 27 patients (92.6%) started eculizumab treatment from the first TMA episode. Nineteen patients (70.4%) had received plasma therapy in the past year before eculizumab treatment. Thirteen patients (48.1%) were receiving dialysis at diagnosis.

The median (range) total duration of eculizumab treatment in aHUS patients at the data cutoff was 51.0 (0–125) weeks. Total duration of eculizumab treatment was < 1 week for 2 patients, ≥ 1–< 4 weeks for 2 patients, ≥ 4–< 26 weeks for 3 patients, and ≥ 26 weeks for 20 patients. The dosing interval was altered for 18 of 27 patients (66.7%), and the dose was reduced for 2 patients (11.1%). At the date of data cutoff, 9 of 27 patients had discontinued eculizumab. Discontinuation of eculizumab was based on the doctor’s decision in 6 patients (improvement in 5 patients, isolation of STEC in 1 patient), death in 3 patients (described below), a family request in 1 patient and an AR in 1 patient (Supplementary Table 4). Three patients who discontinued eculizumab harbored abnormalities in complement genes; a patient who subsequently received a diagnosis of STEC-HUS had variants in *MCP* and *CFB* genes, another patient had anti-CFH antibodies and *CFHR1*/*3* deletion, and the third patient harbored variants in *CFH* and *MCP* (Table 1). No variants were identified in 2 patients. Genetic tests were not performed for the remaining 4 patients.

### Effectiveness of eculizumab in aHUS

Clinical endpoints of effectiveness during eculizumab treatment for pediatric patients are shown in Table 2. TMA event–free status was achieved in 23/27 patients (85.2, 95% confidence interval [CI] 66.3–95.8%). Complete TMA response and hematologic normalization, which were defined as maintenance of hematologic and renal outcomes for 4 weeks, were achieved in 8/22 patients (36.4, 95% CI 17.2–59.3%) and 9/22 patients (40.9, 95% CI 20.7–63.6%), respectively. The overall survival of aHUS patients was 88.4% (*n* = 24), as shown in Supplementary Fig. 1.

**Table 2:** Clinical endpoints of effectiveness for patients with aHUS

TMA event-free status, *n*	27
*n* (%)	23 (85.2)
95%CI	66.3–95.8
Complete TMA response, *n*	22
*n* (%)	8 (36.4)
95% CI	17.2–59.3
Hematologic outcome	
Hematologic normalization, *n*	22
*n* (%)	9 (40.9)
95% CI	20.7–63.6
Platelet count normalization, *n*	23
*n* (%)	18 (78.3)
95% CI	56.3–92.5
LDH normalization, *n*	25
*n* (%)	12 (48.0)
95% CI	27.8–68.7
Hemoglobin improvement ≥ 2 g/dl, *n*	27
*n* (%)	17 (63.0)
95% CI	42.4–80.6
Renal outcome	
Serum creatinine level decrease by ≥ 25%, *n*	27
*n* (%)	18 (66.7)
95% CI	46.0-83.5
eGFR improvement by ≥ 15 ml/min/1.73 m^2^, *n*	12
*n* (%)	9 (75.0)
95% CI	42.8–94.5

eGFR was calculated for pediatric patients aged 2 years through 18 years

*CI* confidence interval, *eGFR* estimated glomerular filtration rate, *LDH* lactate dehydrogenase, *TMA* thrombotic microangiopathy

**Fig. 1:**
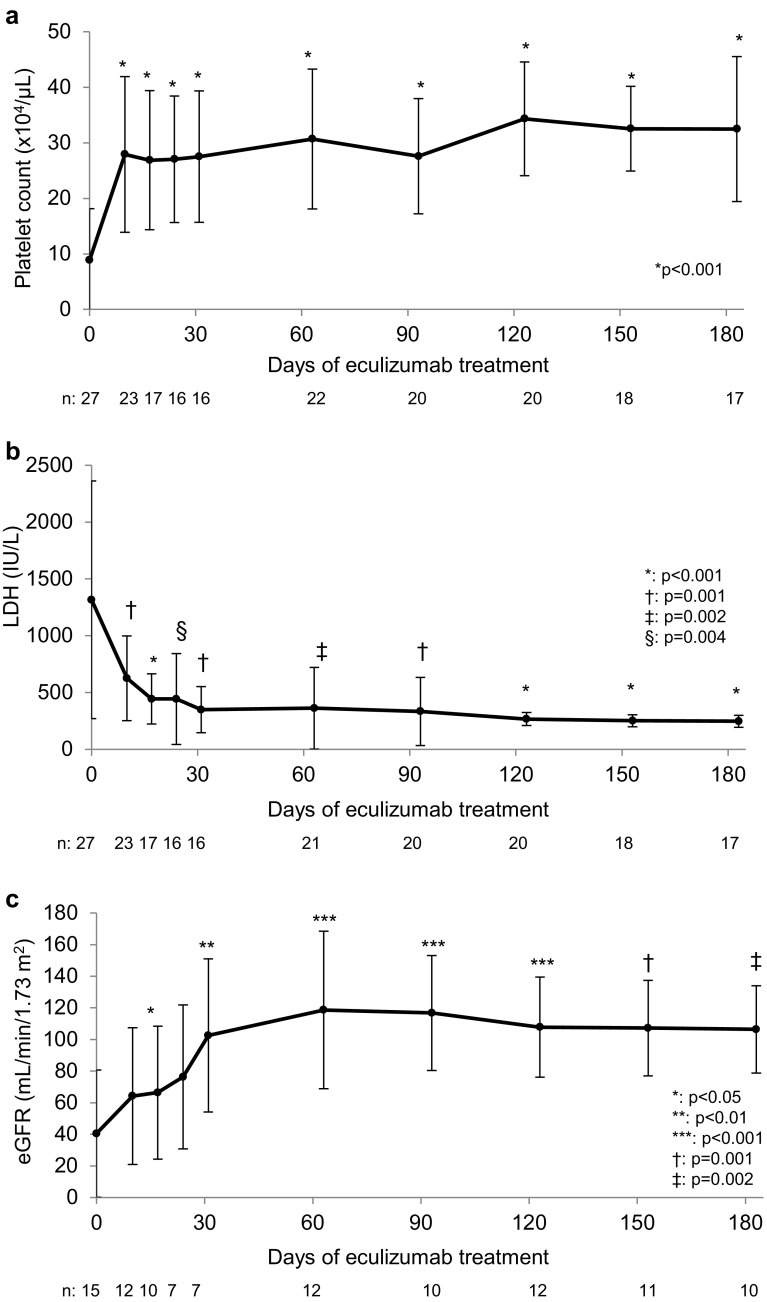
PLT, LDH, and eGFR levels during eculizumab treatment of patients with aHUS. The eGFR for pediatric patients aged 2 years through 18 years was calculated using the following formulas, with “X” as $$\begin{aligned} {\text{body height }}\left( {\text{m}} \right):{\text{ eGFR}}~= ~{\text{11}}0.{\text{2}}~ \times ~[ - ~{\text{1}}.{\text{259}}~ \times ~{\text{5}}~+~{\text{7}}.{\text{815}}~ \times ~{\text{4}}~ - ~{\text{18}}.{\text{57}}~ \times ~{\text{3}}~+~{\text{21}}.{\text{39}}~ \times ~{\text{2}}~ - ~{\text{11}}.{\text{71X}}~+~{\text{2}}.{\text{628}}]/\left( {{\text{serum creatinine}}} \right)~+~{\text{2}}.{\text{93 for boys and 11}}0.{\text{2}}~ \times ~[ - ~{\text{4}}.{\text{536}}~ \times ~{\text{5}}~+~{\text{27}}.{\text{16}}~ \times ~{\text{4}}~ - ~{\text{63}}.{\text{47}}~ \times ~{\text{3}}~+~{\text{72}}.{\text{43}}~ \times ~{\text{2}}~ - ~{\text{4}}0.0{\text{6X}}~+~{\text{8}}.{\text{778]}}/\left( {{\text{serum creatinine}}} \right)~+~{\text{2}}.{\text{93}} \end{aligned}$$ for girls [11]. Although eGFR is generally used to evaluate chronic kidney disease, it was used to assess acute kidney dysfunction in this study. Changes from baseline were compared using the paired *t* test

PLT normalization was achieved in 18/23 patients (78.3, 95% CI 56.3–92.5%) (Table 2). Mean (SD) PLT was 8.89 (9.24) × 10^4^/µL at baseline and 27.9 (14.0) × 10^4^/µL at 10 days (Fig. 1). Mean increase of PLT from baseline to 10 days was 18.6 (15.7) × 10^4^/µL (*P* < 0.001). LDH normalization was achieved in 12/25 patients (48.0, 95% CI 27.8–68.7%). Mean (SD) LDH was 1315 (1045) IU/I at baseline and 349 (202) IU/I at 31 days (Fig. 1). Mean change in LDH from baseline to 31 days was a reduction of 1201 (1185) IU/L (*P* = 0.001), although LDH levels in 92.6% (25/27) of patients had decreased by < 404 IU/L at the end of observation (Supplementary Fig. 2a, b). An eGFR improvement of ≥ 15 ml/min/1.73 m^2^ was achieved in 9/12 patients older than 2 years (75.0, 95% CI 42.8–94.5%). Mean (SD) eGFR was 40.4 (40.3) ml/min/1.73 m^2^ at baseline and 102.5 (48.4) ml/min/1.73 m^2^ at 31 days (Fig. 1). Mean increase in eGFR of individual patients from baseline to 31 days was 79.7 (43.3) ml/min/1.73 m^2^ (*P* = 0.003). Eight of 13 patients (61.5%) receiving dialysis at baseline were able to discontinue dialysis. Of the remaining 5 patients, 2 (1 younger than 2 years) continued dialysis and 3 died.

### Safety analysis of aHUS patients

For the 31 pediatric aHUS patients in the safety analysis set, total exposure time was 33.0 patient-years. Twenty-four ARs (0.73 per patient-year) were reported in 8 patients (Table 3). ARs reported in 2 or more patients were hypertension and medical equipment–related infection (2 patients each, 0.06 per patient-year for each event); the other 20 ARs occurred in 1 patient each (0.03 per patient-year for all). No meningococcal infection was reported during eculizumab treatment or the observation period (Table 3).

**Table 3:** Treatment-emergent adverse reaction in patients with aHUS (*n* = 31)

	Adverse reaction	Serious adverse reaction
	Number of cases (/person-year)	Number of cases (/person-year)
Total exposure time (patient-years)	33.02	
Total number of manifestations	24 (0.73)	10 (0.30)
Medical equipment-related infection	2 (0.06)	2 (0.06)
Hypertension	2 (0.06)	1 (0.03)
Tympanitis	1 (0.03)	0 (0.00)
Acute otitis media	1 (0.03)	0 (0.00)
Peritonitis	1 (0.03)	1 (0.03)
Anal abscess	1 (0.03)	0 (0.00)
Sepsis	1 (0.03)	1 (0.03)
Upper respiratory tract infection	1 (0.03)	0 (0.00)
RS virus infection	1 (0.03)	0 (0.00)
Loss of appetite	1 (0.03)	0 (0.00)
Head discomfort	1 (0.03)	0 (0.00)
Seizures	1 (0.03)	1 (0.03)
Thrombosis	1 (0.03)	1 (0.03)
Pulmonary hemorrhage	1 (0.03)	1 (0.03)
Laryngeal stenosis	1 (0.03)	1 (0.03)
Vomiting	1 (0.03)	0 (0.00)
Hives	1 (0.03)	0 (0.00)
Erythema	1 (0.03)	0 (0.00)
Rash	1 (0.03)	0 (0.00)
Injection site reaction	1 (0.03)	1 (0.03)
Fever	1 (0.03)	0 (0.00)
Malaise	1 (0.03)	0 (0.00)

Ten serious ARs were reported in four patients (0.30 per patient-year). The infection-related serious ARs were medical equipment-related infection (2 patients) and peritonitis and sepsis (1 patient each). One patient twice developed an infusion-site reaction (rash and/or wheeze), but both reactions resolved within 1 day.

The 3 aHUS patients who died were infants younger than 1 year (Supplementary Fig. 1 and Supplementary Table 4). The details of their clinical courses are described in the “Brief Case Report” in Supplementary Materials. In one patient, the AR that possibly resulted in death was pulmonary hemorrhage. The second patient died of acute heart failure and the third died of acute liver failure and exacerbation of renal dysfunction. None of these deaths was judged to be related to eculizumab.

### Patient characteristics and outcomes of TMA caused by underlying disease or complement-amplifying condition (secondary TMA)

This interim analysis of the PMS included 17 patients with secondary TMA, which is referred to as secondary aHUS in other reports [12, 13]. Median age (range) at baseline was 2 (0–17) years, and 5 (29.4%) patients were younger than 2 years. Abnormalities of complement genes were identified in 3 of 5 patients tested. One had *CFHR3-1* heterozygous deletion, another had *CFHR2* heterozygous deletion with anti-CFH antibodies, and the third had homozygous deletion of the *CFHR1* first intron. Table 4 summarizes the underlying diseases and complement-amplifying conditions (CACs) in those 17 patients. Two patients had infection (pneumococcal infection and Bordetella pertussis; *n* = 1 each), 1 had rheumatic disease (systemic lupus erythematosus), and 1 patient had a condition related to drug use. The underlying diseases were unknown in three patients. Ten patients (58.8%) had undergone hematopoietic stem cell transplantation (HSCT).

**Table 4:** Underlying diseases and complement-amplifying conditions in patients with secondary TMA (*n* = 17)

Underlying disease/complement-amplifying conditions	No. of patients	Outcome				
		Improvement of symptoms/TMA	Remission	Insufficient response	Death	Other/unknown
HSCT						
HSCT	6	–	1^a^	1^a^	4 (2)^a^	–
Complement dysregulation^b,c^	2	–	1^a,b^	–	1^c^	–
Drug	1	–	–	–	1^a^	–
Other	1	1^a,d^	–	–	–	–
Infection	2	–	–	1	–	1^d^
Drug	1	–	–	–	1^a^	–
Rheumatic disease	1	–	–	–	–	1^f^
Unknown	3	1^a,g^	1^h^	–	1^a^	–

Hematopoietic stem cell transplantation (HSCT) includes bone marrow (*n* = 5), cord blood (*n* = 3) and autologous peripheral blood stem cells transplant (*n* = 2). Four of 10 patients with HSCT were treated with calcineurin inhibitors (tacrolimus) at least 1 year before eculizumab treatment. Parentheses denote 2 out of 4 patients had a malignant tumor as a complication

^a^Malignancy

^b^Heterozygous deletion of *CFHR3-1*

^c^Heterozygous deletion of *CFHR2* and anti-CFH antibody positive

^d^Engraftment syndrome

^e^Eculizumab treatment was continued

^f^Systemic lupus erythematosus

^g^Homozygous deletion of the *CFHR1* 1st intron

^h^Diagnosed as STEC-HUS afterward

The median (range) total duration of eculizumab treatment was 3.0 (0–101) weeks. Three patients were treated with eculizumab for < 1 week, 6 patients for ≥ 1–< 4 weeks, 7 patients for ≥ 4–< 26 weeks, and 1 patient for ≥ 26 weeks. At the data cutoff, 16–17 patients had discontinued eculizumab therapy. The reason for discontinuation was the doctor’s decision in 9 patients (improvement in 7 patients, STEC-HUS diagnosis in 1 patient, and no description for the last patient). Three patients discontinued because of an insufficient response, 4 patients because of AEs, and 7 patients due to death (Supplementary Table 5). Among the 10 patients with HSCT-associated TMA (HSCT-TMA), 4 survived (3 of which experienced remission or improvement of TMA). Two patients (malignancy and STEC-HUS; *n* = 1 each) showed remission or improvement of TMA.

### Safety analysis in secondary TMA

For the 17 secondary TMA patients, total exposure time was 3.29 patient-years, and 10 ARs (3.04 per patient-year) were reported in 5 patients (Table 5). Each AR occurred once in a patient (0.30 per patient-year for all). Most ARs (9 of 10 events) were considered serious (2.74 per patient-year), and serious ARs were reported in five patients. The infection-related serious ARs were pneumonia, bacteremia, urinary tract infection, and herpes zoster. No meningococcal infection was reported during eculizumab treatment.

**Table 5:** Treatment-emergent adverse reaction in patients with secondary TMA (*n* = 17)

	Adverse reaction	Serious adverse reaction
	Number of cases (/person-year)	Number of cases (/person-year)
Total exposure time (patient-years)	3.29	
Total number of manifestations	10 (3.04)	9 (2.74)
Pneumonia	1 (0.30)	1 (0.30)
Bacteremia	1 (0.30)	1 (0.30)
Urinary tract infection	1 (0.30)	1 (0.30)
Herpes zoster	1 (0.30)	1 (0.30)
Leukopenia	1 (0.30)	1 (0.30)
Hypertension	1 (0.30)	1 (0.30)
Pulmonary edema	1 (0.30)	1 (0.30)
Hives	1 (0.30)	0 (0.00)
Renal dysfunction	1 (0.30)	1 (0.30)
Fever	1 (0.30)	1 (0.30)

Eight patients with secondary TMA died, and 5 of these 8 patients (62.5%) had a malignancy. Six (75%) had HSCT-TMA, and half (3/6) of those with HSCT-TMA died after 1 or 2 doses of eculizumab. AEs that led to discontinuation of eculizumab therapy were progression of neuroblastoma, multi-organ failure, adenovirus infection, cerebral hemorrhage, and progression of EBV-associated lymphoproliferative disease. Seven deaths were judged to be unrelated to eculizumab. An AR related to death (leukopenia) was noted in 1 patient, as described in the “Brief Case Report” in the Supplementary Information.

## Discussion

Few retrospective and prospective clinical studies have evaluated the safety and efficacy of eculizumab in Japanese patients with aHUS [5, 6, 14–17]. The outcomes in this aHUS cohort are consistent with those reported in previous clinical study [17] and showed improved TMA event-free status in Japanese pediatric patients treated with eculizumab. In a pivotal study of pediatric patients, the endpoints of TMA event-free status and PLT normalization were achieved in 95% of patients [17]. In the present analysis, 78 and 85% of patients, respectively, achieved these clinically relevant endpoints.

In a previous pediatric trial, the endpoint of complete TMA response was achieved in 64% of pediatric patients [17]. Despite successful achievement of TMA event-free status, complete TMA response was achieved in 36% of the present patients. Complete TMA response indicates improvement in the entire triad of PLT, LDH, and creatinine. Creatinine level decreased in 67% of patients, but LDH normalization was seen in fewer than half of patients. Both group and individual patient data showed that although LDH level clearly decreased within a few weeks, it did not reach the age-matched upper limit of normal in some patients, perhaps because of technical problems such as difference in institutional standards and hemolysis at blood sampling for a small child.

Existing evidence indicates that eculizumab is well-tolerated and that ARs are uncommon [5]. In this report, three infants with aHUS died. However, none of these deaths were related to eculizumab. A previous study suggests that 3% of Japanese population are nonresponders of eculizumab because of a specific C5 polymorphism leading to structural change of the C5 binding site of eculizumab [18]. Unfortunately, this polymorphism was not investigated in the present study.

Despite eculizumab treatment, patients with secondary TMA had poor outcomes. Eight of 17 patients died during the observation period. Most of these patients had underlying HSCT-TMA or malignancies: 4 of 10 patients with HSCT-TMA (40%) survived and 3 showed evidence of TMA improvement. A previous prospective observational study of HSCT-TMA showed that overall survival was significantly longer for pediatric patients treated with eculizumab than for those who did not receive eculizumab (62 vs. 9%, respectively, at 1 year after TMA diagnosis) [19, 20]. However, in that study eculizumab dose was increased significantly, which was not the case in this PMS study. Additional evidence will be required to determine whether adjustment of the timing and regimen for eculizumab administration improves outcomes for patients with HSCT-TMA [19, 20].

The Japanese Society of Nephrology has issued precautions for use of eculizumab in patients with secondary TMA [21], which is classified according to underlying conditions or CACs [10, 22]. Findings from a retrospective study suggest that the benefits of eculizumab vary in relation to the underlying condition of secondary aHUS [22]. The present study could not address the effectiveness of eculizumab for each subgroup because of the small number of patients in each category, although five patients experienced remission or improvement of TMA. Thus, the benefit of eculizumab in secondary TMA should be carefully evaluated in future studies.

Mechanism of action of eculizumab may increase the risk of infection by encapsulated bacterial organisms, particularly *Neisseria meningitidis* from 1000-fold to 2000-fold compared to normal population [5, 23]. Although anti-meningococcal vaccination is mandatory before the first dose of eculizumab except in the case of urgent treatment, meningococcal infection could occur even in patients who have received the vaccination [23]. In this study, none of the 13 infection-related ARs in aHUS and secondary TMA was related to *N. meningitides*. Importantly, no predominant pathogen was identified. However, one patient with neisserial infection, who fully recovered with appropriate antibiotic treatment, was reported after the data cutoff. In addition, in this study despite the mandatory requirement for vaccination, the percentage of meningococcal vaccination was relatively low (54.2%). An increased vigilance and education of the risk of meningococcal infection as a high-mortality disease and the requirement for vaccination will need to be put in place for patients treated with eculizumab in Japan.

This interim analysis had some limitations, including missing data and inadequate or incomplete follow-up, which resulted in variable patient numbers for assessments. Moreover, interpretation of disease characteristics and ARs by treating physicians may be inconsistent. As this was conducted in a clinical practice setting, there was no control group and greater variability in patient background, medical practice and treatment, (notably treatment duration was short and dosing was not as per approved regimen in a large proportion of patients) and follow-up schedule. Therefore, the results should be carefully interpreted.

In conclusion, this interim analysis confirmed that eculizumab is well-tolerated and effective for Japanese pediatric patients with aHUS in a real-world setting.

## Electronic supplementary material

Below is the link to the electronic supplementary material.

Supplementary material 1 (DOC 79 KB)

Supplementary material 2 (PPTX 99 KB)
