# Supplementary material for: Safety and effectiveness of eculizumab for pediatric patients with atypical hemolytic–uremic syndrome in Japan: interim analysis of post-marketing surveillance
Source: Clin Exp Nephrol. 2018 Jul 23;23(1):112–21. doi: 10.1007/s10157-018-1610-2 (PMC6344608; doi:10.1007/s10157-018-1610-2)
Supplement: Supplementary file 1 — Supplementary material 1 (DOC 79 KB) [file 10157_2018_1610_MOESM1_ESM.doc]

**Supplementary Materials**

**Supplementary Table 1** Dosing schedule of eculizumab for pediatric patients

* Anti-meningococcal vaccination is mandatory before the first dose of eculizumab [1]. Since this is a real-world study, actual dosing and duration of treatment were determined by patients' physicians.

**Supplementary Table 2** Definitions of clinical endpoints of effectiveness and adverse events/reactions

**Supplementary Table 3** Genetic variants and allele frequencies

**Supplementary Table 4** Continuation/discontinuation status of patients with aHUS treated with eculizumab

Note: * The clinical courses of these 3 patients are described in the “Brief Case Report” section (patients 1, 2, and 3).

**Supplementary Table 5** Continuation/discontinuation status of patients with secondary TMA treated with eculizumab

**Supplementary Fig. 1** Kaplan–Meier survival curve for patients with aHUS

Kaplan–Meier analysis was used to calculate time-to-event values for overall survival from the date of first administration of eculizumab to the date of occurrence of the events.

**Supplementary Fig. 2 (a, b)** LDH levels during eculizumab treatment for individual patients with aHUS

* in (a), Patients with an increase in LHD at the end of the observation period.

**Brief Case Report**

Patient 1 (aHUS):

An 11-week-old boy developed TMA, followed by exacerbation of AKI, acute respiratory failure, and disseminated intravascular coagulation (DIC). The patient was started on eculizumab 7 days after onset of the TMA but died 3 days after the first dose of eculizumab. A possible cause of death was pulmonary hemorrhage, but no obvious relationship was identified between death and eculizumab treatment; DIC was another potential cause of death. Complement gene testing was not performed.

Patient 2 (aHUS):

An 8-month-old boy with progeria had TMA complicated by hypertension and renal dysfunction. He was treated with plasma infusion for 3 days and then started on eculizumab. He developed acute heart failure caused by TMA and died 18 days after the fifth dose of eculizumab. A possible cause of death was heart failure caused by progression of progeria. The death was judged to be unrelated to eculizumab. Complement gene testing was not performed.

Patient 3 (aHUS):

A 6-week-old boy had TMA with acute liver failure complicated by upper respiratory and pulmonary hemorrhage, respiratory failure, and acute kidney injury. He was treated with eculizumab 12 days after onset of TMA. Two days after the first eculizumab dose, he died of acute liver failure complicated by TMA. Complement gene testing was not performed.

Patient 4 (secondary TMA):

A 6-year-old boy with progressive acute myeloid leukemia presented with TMA after bone marrow transplantation and was started on eculizumab 548 days after the TMA manifestation. He received 2 doses of eculizumab but treatment was later discontinued for 37 days because of bacteremia. Eculizumab treatment was restarted with 2 doses. The patient developed leukopenia complicated by pneumonia (adenovirus infection). He died of pneumonia 3 days after the last dose of eculizumab. The causal relationship between eculizumab and pneumonia was described as “unknown”.

**Reference**

1 Soliris package insert version 8. In: Soliris website. http://www.soliris.jp/common/pdf/Soliris_Tempu_Bunsho_08.pdf. Accessed 19 Dec 2017. Japanese.

2 Matsumoto T, Fan X, Ishikawa E, Ito M, Amano K, Toyoda H, et al. Analysis of patients with atypical hemolytic uremic syndrome treated at the Mie University Hospital: concentration of C3 p.I1157T mutation. Int J Hematol. 2014; 100:437–42.

3 Manuelian T, Hellwage J, Meri S, Caprioli J, Noris M, Heinen S, et al. Mutations in factor H reduce binding affinity to C3b and heparin and surface attachment to endothelial cells in hemolytic uremic syndrome. J Clin Invest. 2003; 111:1181–90.

4 Miyata T, Uchida Y, Ohta T, Urayama K, Yoshida Y, Fujimura Y. Atypical haemolytic uraemic syndrome in a Japanese patient with DGKE genetic mutations. Thromb Haemost. 2015; 114:862–3.
