# Supplementary material for: Safety and effectiveness of eculizumab for pediatric patients with atypical hemolytic–uremic syndrome in Japan: interim analysis of post-marketing surveillance
Source: Clin Exp Nephrol. 2018 Jul 23;23(1):112–21. doi: 10.1007/s10157-018-1610-2 (PMC6344608; doi:10.1007/s10157-018-1610-2)
Supplement: Supplementary file 2 — Supplementary material 2 (PPTX 99 KB) [file 10157_2018_1610_MOESM2_ESM.pptx]

## Slide 1
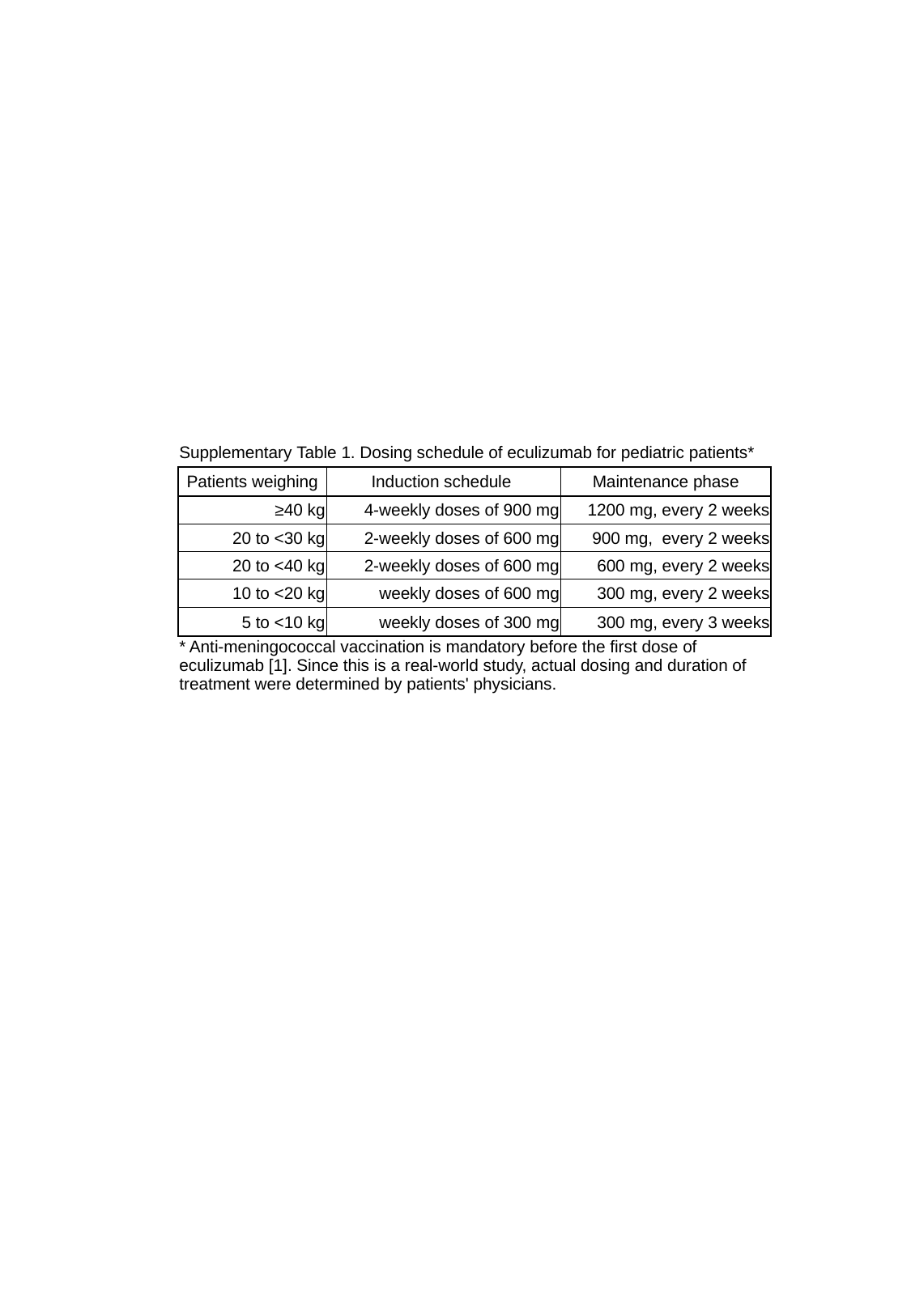

| Supplementary Table 1. Dosing schedule of eculizumab for pediatric patients\* | | |
| --- | --- | --- |
| Patients weighing | Induction schedule | Maintenance phase |
| ≥40 kg | 4-weekly doses of 900 mg | 1200 mg, every 2 weeks |
| 20 to <30 kg | 2-weekly doses of 600 mg | 900 mg, every 2 weeks |
| 20 to <40 kg | 2-weekly doses of 600 mg | 600 mg, every 2 weeks |
| 10 to <20 kg | weekly doses of 600 mg | 300 mg, every 2 weeks |
| 5 to <10 kg | weekly doses of 300 mg | 300 mg, every 3 weeks |
| \* Anti-meningococcal vaccination is mandatory before the first dose of eculizumab [1]. Since this is a real-world study, actual dosing and duration of treatment were determined by patients' physicians. | | |

## Slide 2
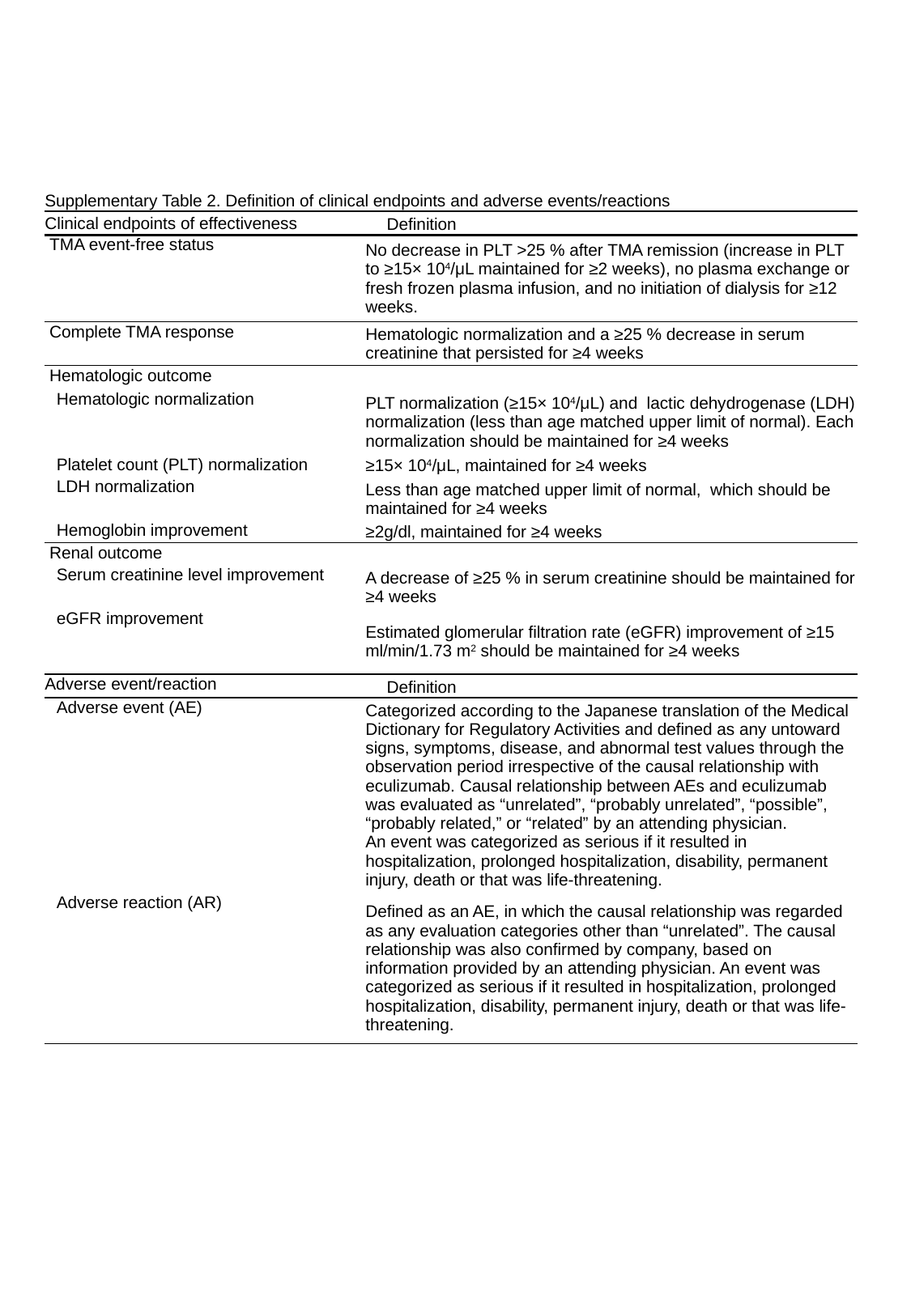

| Supplementary Table 2. Definition of clinical endpoints and adverse events/reactions | |
| --- | --- |
| Clinical endpoints of effectiveness | Definition |
| TMA event-free status | No decrease in PLT >25 % after TMA remission (increase in PLT to ≥15× 104/μL maintained for ≥2 weeks), no plasma exchange or fresh frozen plasma infusion, and no initiation of dialysis for ≥12 weeks. |
| Complete TMA response | Hematologic normalization and a ≥25 % decrease in serum creatinine that persisted for ≥4 weeks |
| Hematologic outcome | |
| Hematologic normalization | PLT normalization (≥15× 104/μL) and lactic dehydrogenase (LDH) normalization (less than age matched upper limit of normal). Each normalization should be maintained for ≥4 weeks |
| Platelet count (PLT) normalization | ≥15× 104/μL, maintained for ≥4 weeks |
| LDH normalization | Less than age matched upper limit of normal, which should be maintained for ≥4 weeks |
| Hemoglobin improvement | ≥2g/dl, maintained for ≥4 weeks |
| Renal outcome | |
| Serum creatinine level improvement | A decrease of ≥25 % in serum creatinine should be maintained for ≥4 weeks |
| eGFR improvement | Estimated glomerular filtration rate (eGFR) improvement of ≥15 ml/min/1.73 m2 should be maintained for ≥4 weeks |
| Adverse event/reaction | Definition |
| Adverse event (AE) | Categorized according to the Japanese translation of the Medical Dictionary for Regulatory Activities and defined as any untoward signs, symptoms, disease, and abnormal test values through the observation period irrespective of the causal relationship with eculizumab. Causal relationship between AEs and eculizumab was evaluated as “unrelated”, “probably unrelated”, “possible”, “probably related,” or “related” by an attending physician. An event was categorized as serious if it resulted in hospitalization, prolonged hospitalization, disability, permanent injury, death or that was life-threatening. |
| Adverse reaction (AR) | Defined as an AE, in which the causal relationship was regarded as any evaluation categories other than “unrelated”. The causal relationship was also confirmed by company, based on information provided by an attending physician. An event was categorized as serious if it resulted in hospitalization, prolonged hospitalization, disability, permanent injury, death or that was life-threatening. |

## Slide 3
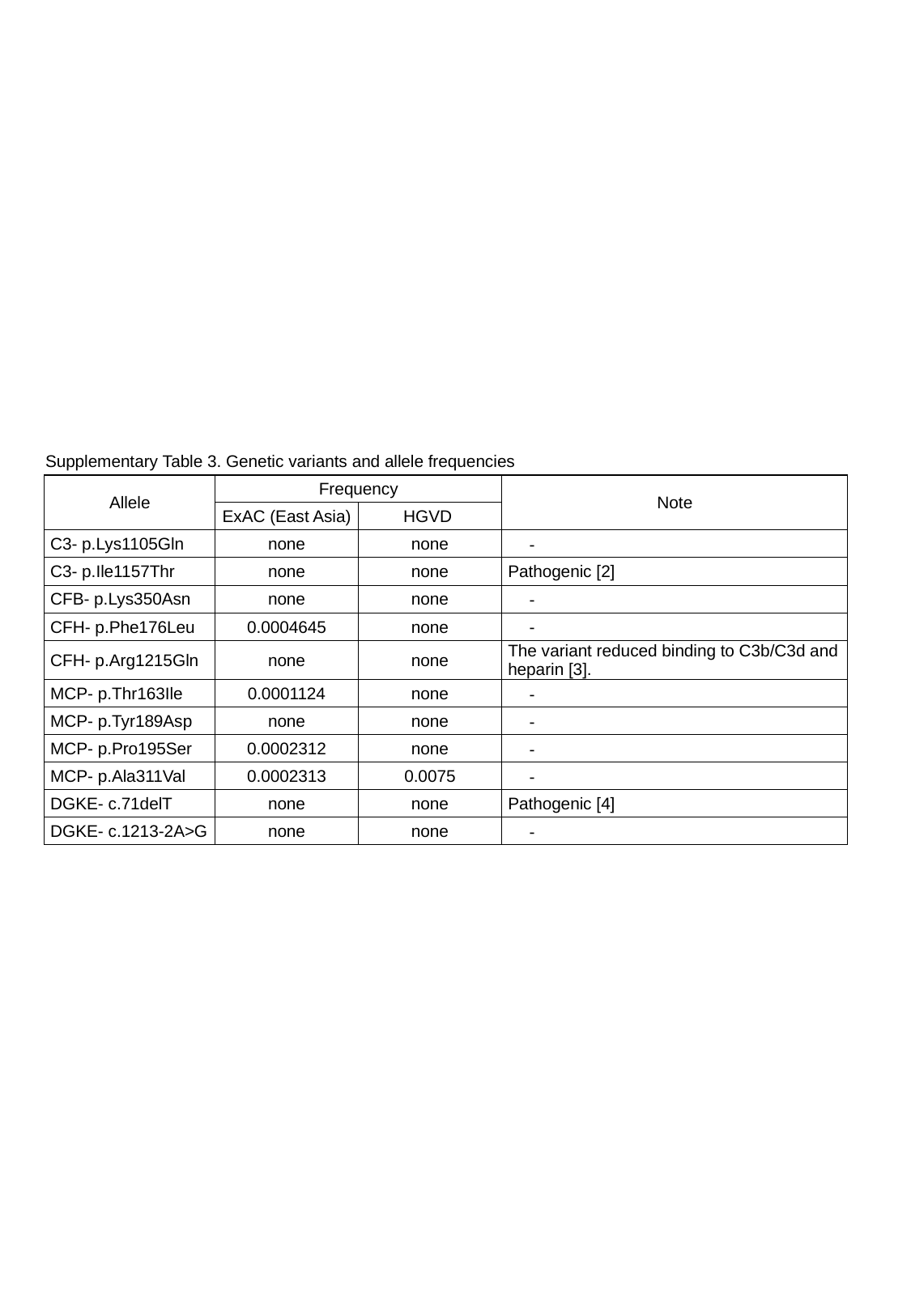

| Supplementary Table 3. Genetic variants and allele frequencies | | | |
| --- | --- | --- | --- |
| Allele | Frequency | | Note |
| | ExAC (East Asia) | HGVD | |
| C3- p.Lys1105Gln | none | none | - |
| C3- p.Ile1157Thr | none | none | Pathogenic [2] |
| CFB- p.Lys350Asn | none | none | - |
| CFH- p.Phe176Leu | 0.0004645 | none | - |
| CFH- p.Arg1215Gln | none | none | The variant reduced binding to C3b/C3d and heparin [3]. |
| MCP- p.Thr163Ile | 0.0001124 | none | - |
| MCP- p.Tyr189Asp | none | none | - |
| MCP- p.Pro195Ser | 0.0002312 | none | - |
| MCP- p.Ala311Val | 0.0002313 | 0.0075 | - |
| DGKE- c.71delT | none | none | Pathogenic [4] |
| DGKE- c.1213-2A>G | none | none | - |

## Slide 4
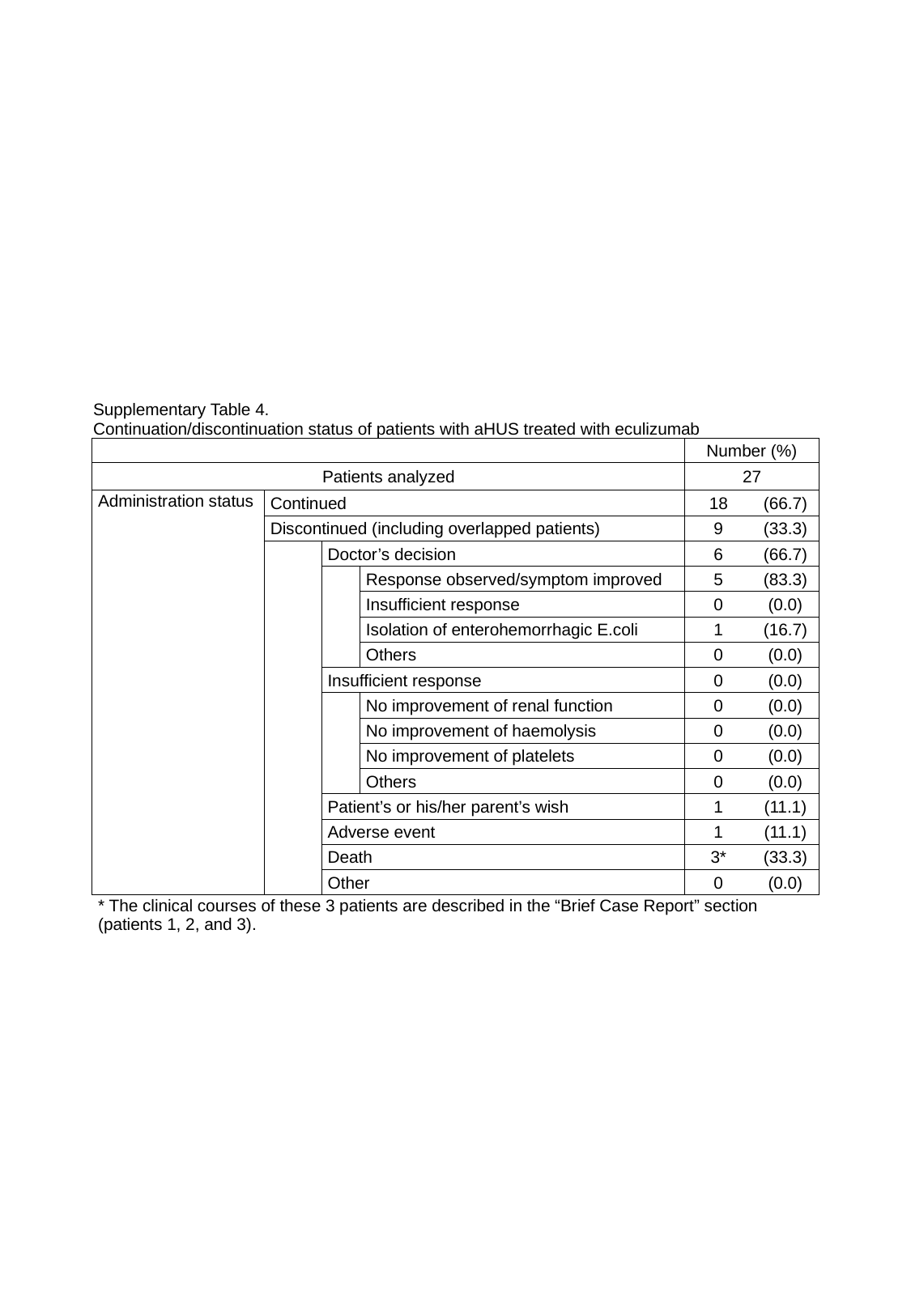

| Supplementary Table 4. Continuation/discontinuation status of patients with aHUS treated with eculizumab | | | | | |
| --- | --- | --- | --- | --- | --- |
| | | | | Number (%) | |
| Patients analyzed | | | | 27 | |
| Administration status | Continued | | | 18 | (66.7) |
| | Discontinued (including overlapped patients) | | | 9 | (33.3) |
| | | Doctor’s decision | | 6 | (66.7) |
| | | | Response observed/symptom improved | 5 | (83.3) |
| | | | Insufficient response | 0 | (0.0) |
| | | | Isolation of enterohemorrhagic E.coli | 1 | (16.7) |
| | | | Others | 0 | (0.0) |
| | | Insufficient response | | 0 | (0.0) |
| | | | No improvement of renal function | 0 | (0.0) |
| | | | No improvement of haemolysis | 0 | (0.0) |
| | | | No improvement of platelets | 0 | (0.0) |
| | | | Others | 0 | (0.0) |
| | | Patient’s or his/her parent’s wish | | 1 | (11.1) |
| | | Adverse event | | 1 | (11.1) |
| | | Death | | 3\* | (33.3) |
| | | Other | | 0 | (0.0) |
| \* The clinical courses of these 3 patients are described in the “Brief Case Report” section (patients 1, 2, and 3). | | | | | |

## Slide 5
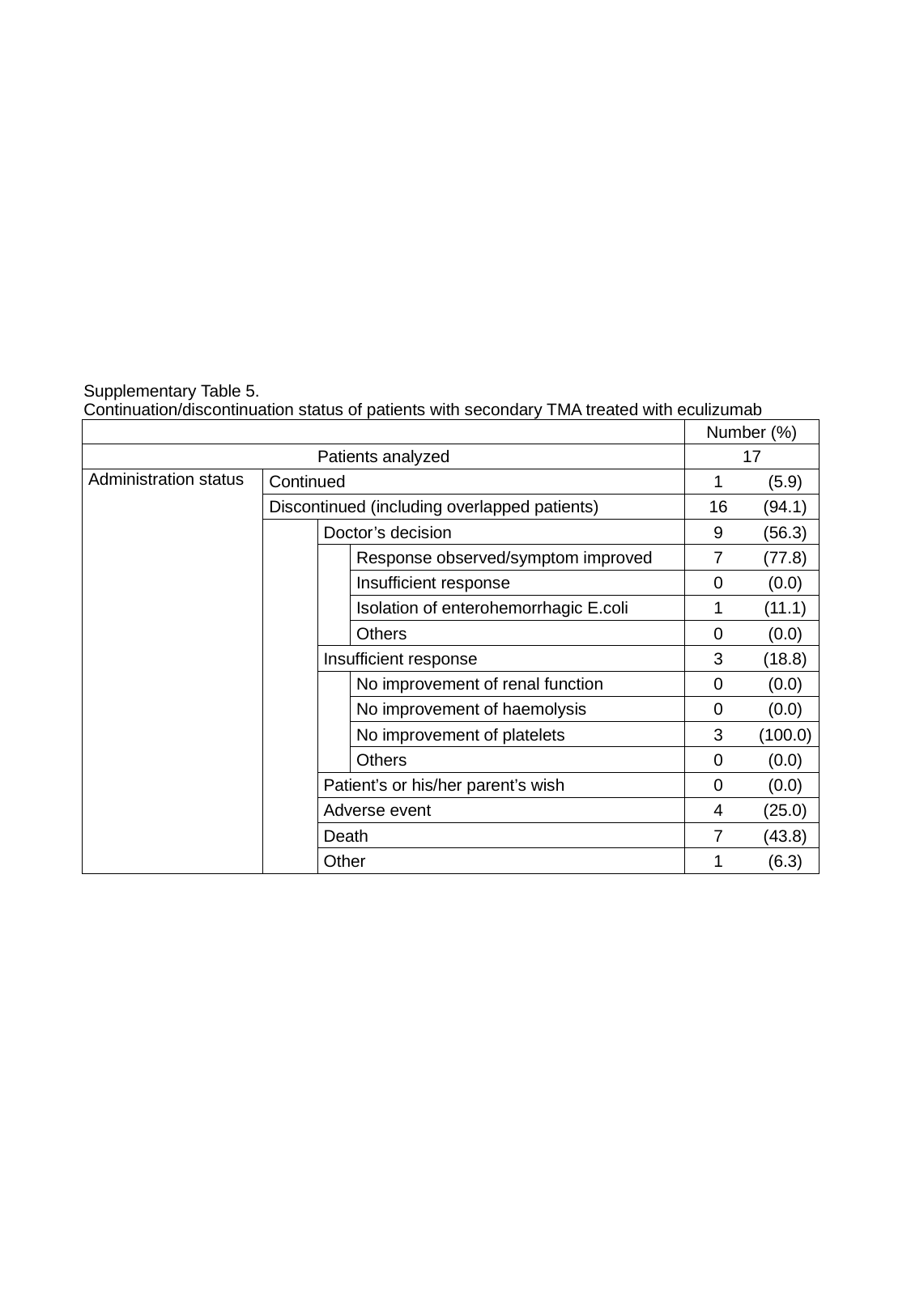

| Supplementary Table 5. Continuation/discontinuation status of patients with secondary TMA treated with eculizumab | | | | | |
| --- | --- | --- | --- | --- | --- |
| | | | | Number (%) | |
| Patients analyzed | | | | 17 | |
| Administration status | Continued | | | 1 | (5.9) |
| | Discontinued (including overlapped patients) | | | 16 | (94.1) |
| | | Doctor’s decision | | 9 | (56.3) |
| | | | Response observed/symptom improved | 7 | (77.8) |
| | | | Insufficient response | 0 | (0.0) |
| | | | Isolation of enterohemorrhagic E.coli | 1 | (11.1) |
| | | | Others | 0 | (0.0) |
| | | Insufficient response | | 3 | (18.8) |
| | | | No improvement of renal function | 0 | (0.0) |
| | | | No improvement of haemolysis | 0 | (0.0) |
| | | | No improvement of platelets | 3 | (100.0) |
| | | | Others | 0 | (0.0) |
| | | Patient’s or his/her parent’s wish | | 0 | (0.0) |
| | | Adverse event | | 4 | (25.0) |
| | | Death | | 7 | (43.8) |
| | | Other | | 1 | (6.3) |

## Slide 6
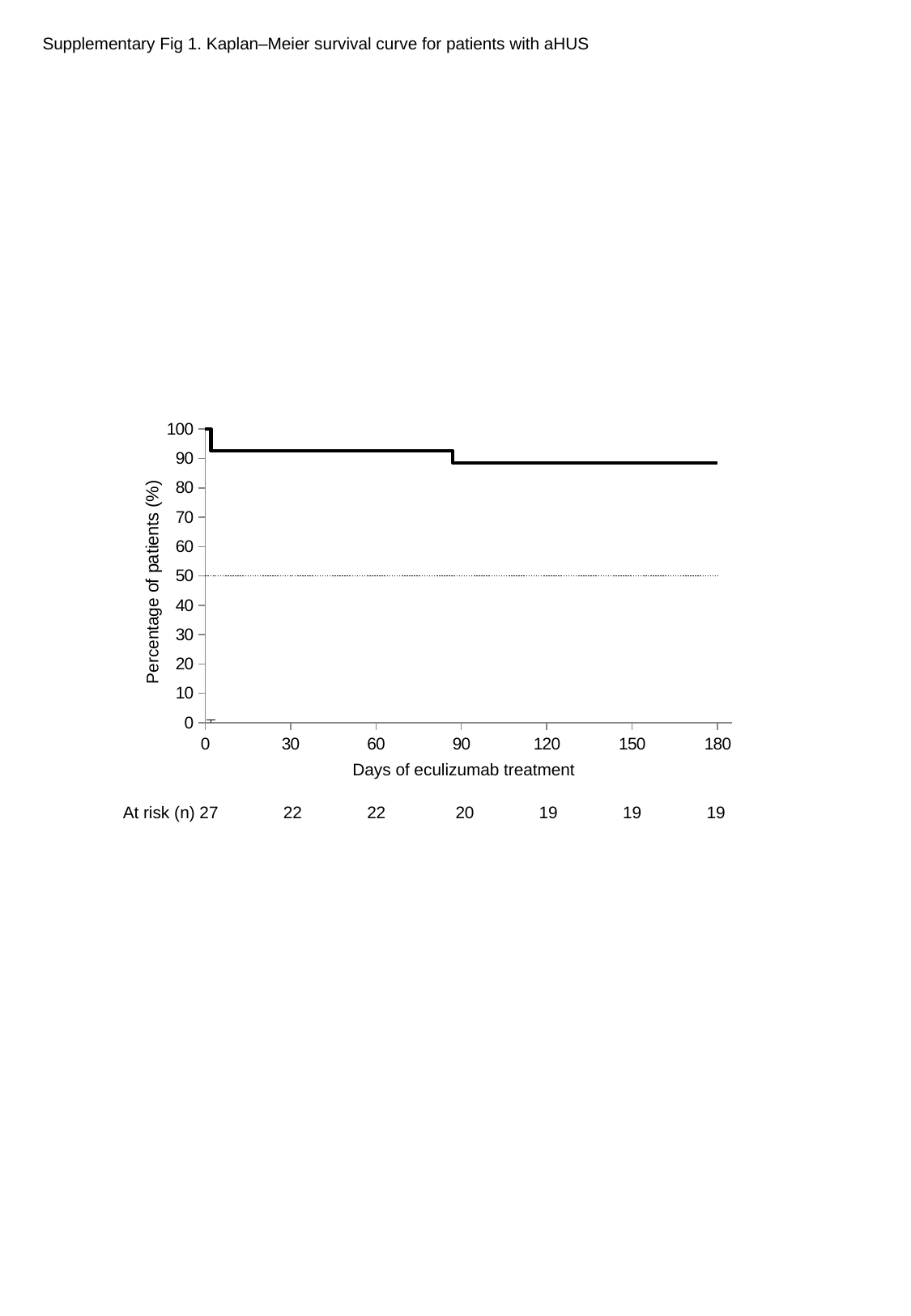

Supplementary Fig 1. Kaplan–Meier survival curve for patients with aHUS
### Chart
| Category | 小児(初回投与時18歳未満) | 0：イベント発現
1：打ち切り(小児) | 50%線 |
|---|---|---|---|Percentage of patients (%)
Days of eculizumab treatment
At risk (n) 27 22 22 20 19 19 19

## Slide 7
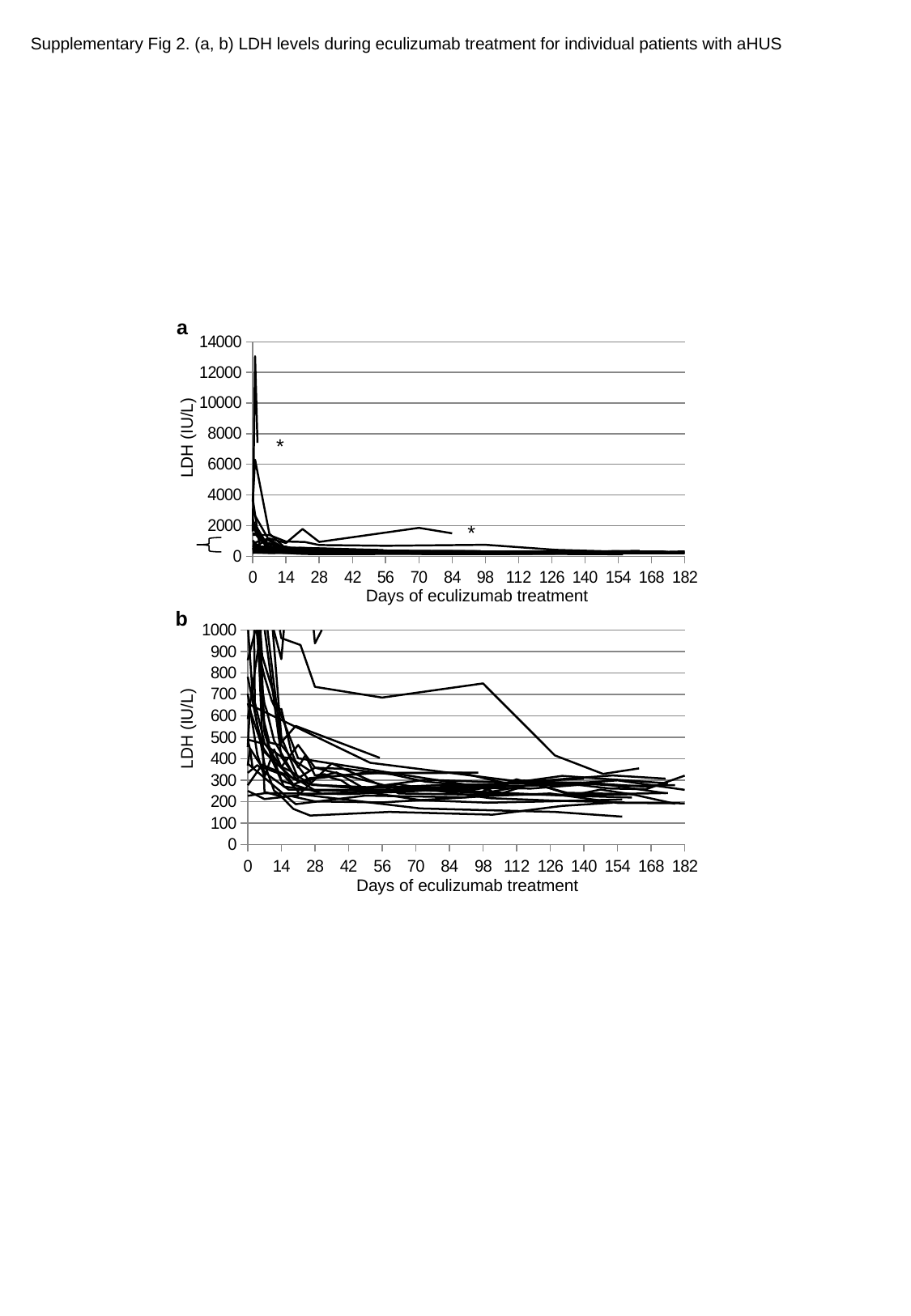

Supplementary Fig 2. (a, b) LDH levels during eculizumab treatment for individual patients with aHUS
a
### Chart
| Category | 測定値 |
|---|---|LDH (IU/L)
*
*
b
Days of eculizumab treatment
b
### Chart
| Category | 測定値 |
|---|---|LDH (IU/L)
Days of eculizumab treatment
